# Adaptafood: an intelligent system to adapt recipes to specialised diets and healthy lifestyles

**DOI:** 10.1007/s00530-025-01667-y

**Published:** 2025-02-01

**Authors:** Andrea Morales-Garzón, Karel Gutiérrez-Batista, Maria J. Martin-Bautista

**Affiliations:** Department of Computer Science and Artificial Intelligence, CITIC-UGR (Research Center for Information and Communication Technologies), University of Granada, Granada, Spain

**Keywords:** Food computing, Recipe adaptation, Word embedding, Healthy diet, Natural language processing, Heterogeneous sources

## Abstract

This paper presents AdaptaFood, a system to adapt recipes to specific dietary constraints. This is a common societal issue due to various dietary needs arising from medical conditions, allergies, or nutritional preferences. AdaptaFood provides recipe adaptations from two inputs: a recipe image (a fine-tuned image-captioning model allows us to extract the ingredients) or a recipe object (we extract the ingredients from the recipe features). For the adaptation, we propose to use an attention-based language sentence model based on BERT to learn the semantics of the ingredients and, therefore, discover the hidden relations among them. Specifically, we use them to perform two tasks: (1) align the food items from several sources to expand recipe information; (2) use the semantic features embedded in the representation vector to detect potential food substitutes for the ingredients. The results show that the model successfully learns domain-specific knowledge after re-training it to the food computing domain. Combining this acquired knowledge with the adopted strategy for sentence representation and food replacement enables the generation of high-quality recipe versions and dealing with the heterogeneity of different-origin food data.

## Introduction

**Fig. 1:**
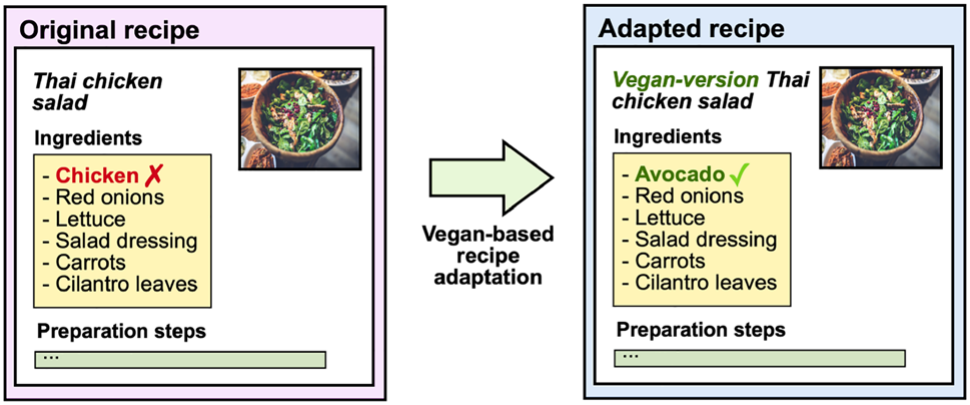
A recipe adaptation for vegan dietary restrictions. A vegan version of the original recipe would change the not-allowed foods from the original recipe (in red), changing it to others that suit the recipe (in green)

The need to adapt food diets and menus for healthier lifestyles is a tangible reality. More and more people are following eco-friendly diets or have restrictions imposed on their daily menus to improve their life expectancy. A healthy diet is essential to prevent and delay the onset of food-related diseases, such as diabetes. The World Health Organisation (WHO) estimates that diabetes is the ninth leading cause of death, with an estimated 1.5 million deaths directly caused by this disease.^1^ A study from Oxford University also cites that swapping to diets rich in fruit and vegetables could save 8 million lives by 2050, reduce gas emissions by two-thirds and avoid climate damages of $1.5 trillion.^2^ Diabetes, high blood pressure or high cholesterol are just some examples of diseases that demand dietary restrictions. People are aware of this, and there is an upward trend to try to change to better habits. One way to integrate good habits into diets is to incorporate small recipe changes to obtain more nourishing and healthier versions. This task, known as recipe adaptation (see Fig. 1), is difficult to automate on a massive scale. It is mainly due to the intrinsic relationships amongst foodstuffs, including smells, tastes, flavours, and regional and local customs. Recipe adaptation is not only a day-to-day need of the population. Automating it would also assist nutritionists in adapting existing diets to the specific needs of their patients. In this context, the Stance4Health project^3^ aims to develop an intelligent nutritionist tool to recommend healthy diets to the population.

Recipe adaptation requires an in-depth study of the existing relationships and patterns of foods in recipes. In these terms, recipe preparation texts are a powerful resource because they contain information about how the ingredients are combined and prepared. These data can help provide better food representations to detect potential ingredient substitutions.

The advent of food computing solutions based on deep learning and the latest advances in natural language processing has opened up a wide range of possibilities to address population health issues related to food [1, 2]. Indeed, language models based on neural networks have demonstrated their effectiveness for learning food attributes in recipes [3], thus allowing modelling recipes and representing their features and food relationships. In a previous study, we demonstrated that language models not based on attention networks show similar behaviour, with it being difficult to take a big step to improve their results for the recipe adaptation task [4]. Additionally, non-dynamic embeddings pose a problem in food computing, as cooks may use the same ingredient for many different purposes (e.g., we can use flour for baking a cake or frying a fish). This fact motivates a need for food text description embeddings that can differentiate between different kinds of courses and cooking styles. The recipe adaptation task requires dealing with the fact that most online-available recipe datasets do not include specialised nutrition information about their ingredients. Consequently, it requires a prior data alignment between recipes, and specialised nutrition datasets are required to extrapolate ingredient data to the recipe level. It involves the further need to deal with heterogeneous data since recipe data and nutrition facts (i.e., nutritional content regarding macro and micro-nutrients in food packaging) are usually obtained from sources of different origins. On the other hand, some datasets only include image-based information regarding the recipes. In these cases, it is essential to develop robust tools to deal with heterogeneous data coming from images and/or texts.

To address the above limitations, in this paper, we propose a multimodal framework for recipe adaptation, considering a list of restrictions. To deal with contextual data, we use attention mechanisms as the basis of the adaptation engine to consider contextual data when training the model. Specifically, we have used the learned features to generate food description embeddings to perform two similarity/semantic tasks: (1) align the embeddings of food items from both the recipe and specialised nutrition datasets to expand recipe information with nutrition facts regarding their ingredients; (2) use the semantic features embedded in the representation vector to detect food substitutes for the ingredients in the recipe.

### Main contributions

**Fig. 2:**
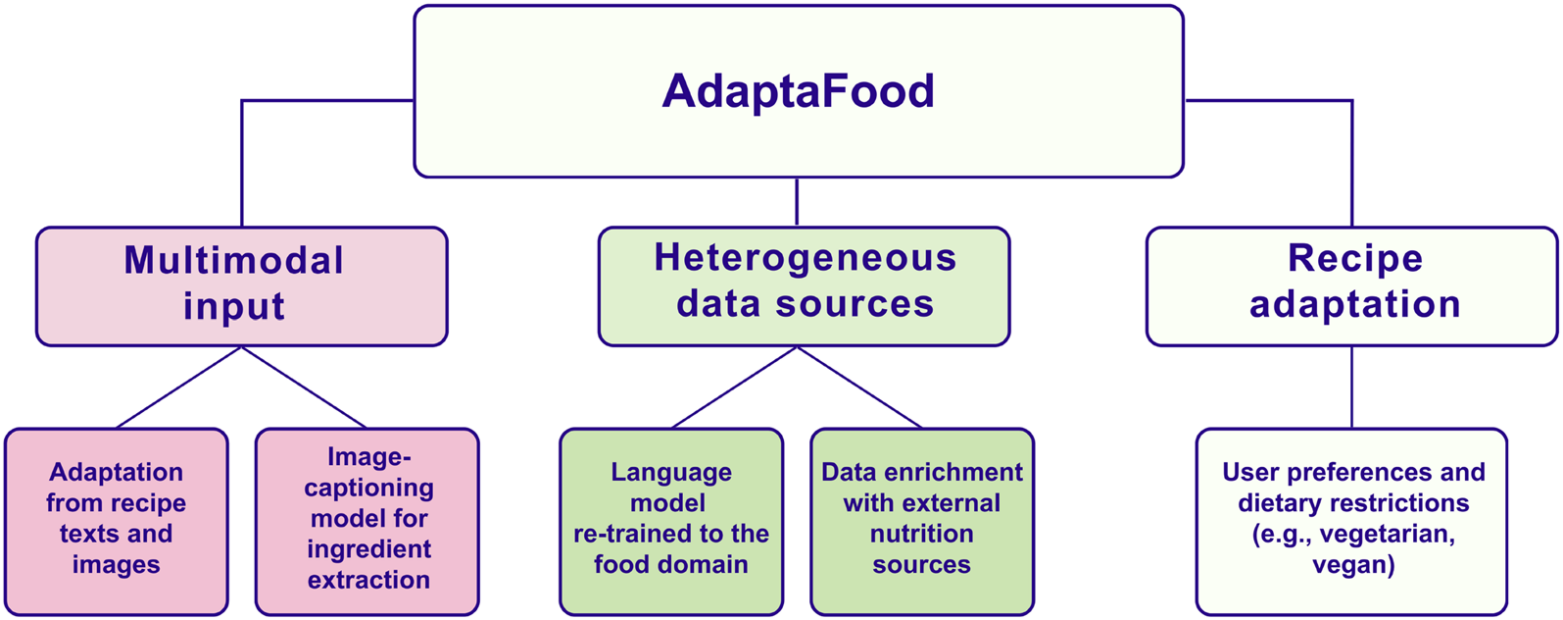
Main contributions of AdaptaFood (highlighted in pink and green colour)

**Fig. 3:**
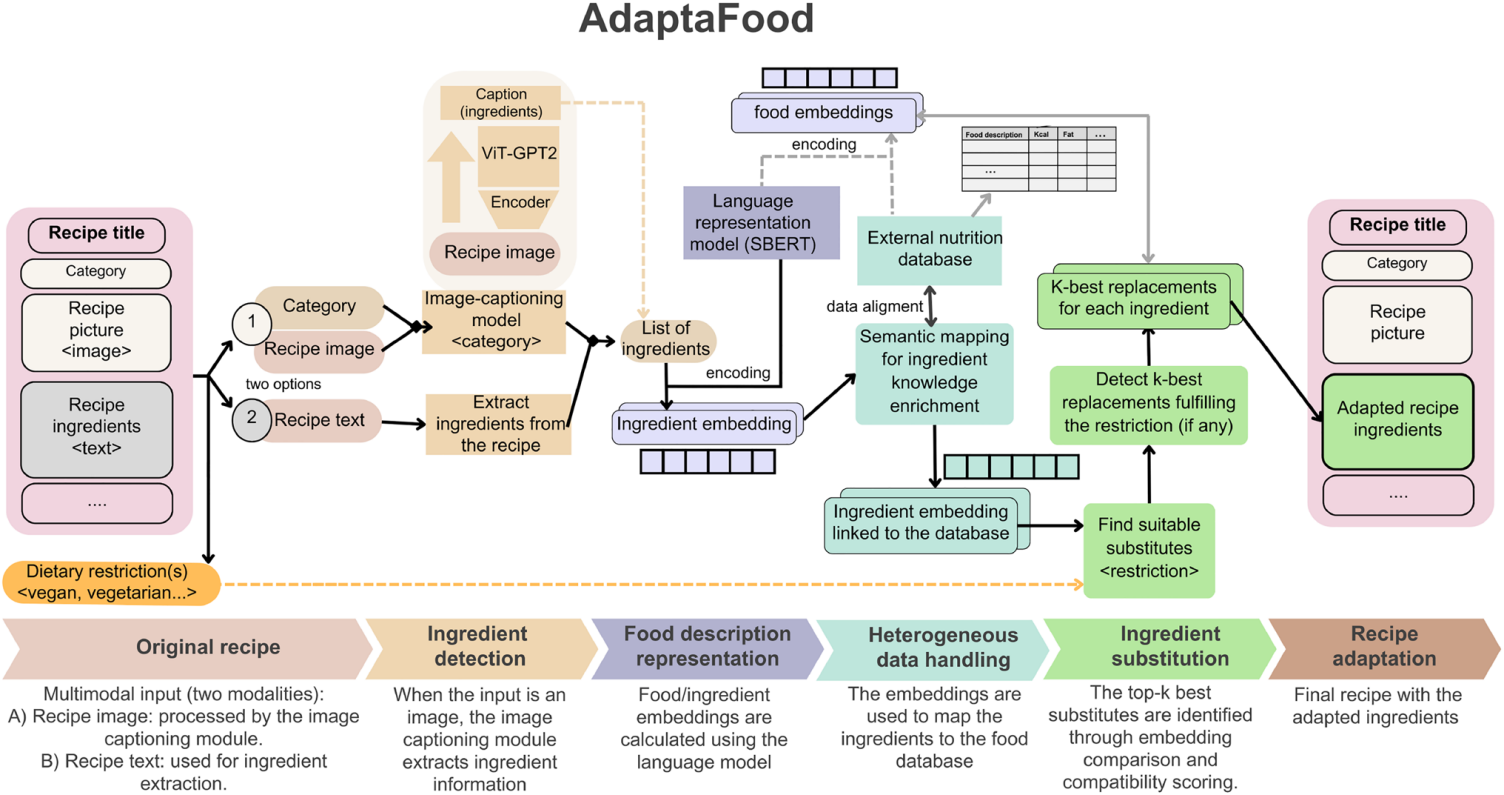
Overview of the proposed approach to adapt a food recipe based on one or more dietary restrictions. The system accepts two types of inputs: a recipe image or text. The bottom section of the figure highlights the input, methods, and output of AdaptaFood, with process colours corresponding to the relevant information in the flowchart

We present **AdaptaFood** (Fig. 3), a food computing system for obtaining personalised recipe versions considering ingredient restrictions.^4^ The main contributions of this study are as follows:

1. A multimodal recipe adaptation approach that allows us to adapt recipes to dietary restrictions and healthy preferences from images and textual data (see blocks in pink in Fig. 2).
2. A sentence-based domain-specific BERT model for food-related problems, obtained by re-training BERT on recipe instructions. This model, enriched with specialised nutritional information, generates recipe versions that satisfy a given list of restrictions (see blocks in green in Fig. 2).
3. An in-depth study of the relevance of word-level token embeddings when building sentence-level based models. We have analysed the role of the token embedding layers (and their aggregation) in the quality of the sentence representations of the food descriptions.
4. Finally, we provide the research community with a competitive approach that achieves remarkable results, with a user satisfaction score of 4 out of 5 on average.

As far as we know, this is the first recipe adaptation approach that can deal with text and image inputs. In addition, AdaptaFood allows us to integrate specialised nutrition information from external knowledge sources for ingredient substitution, distinguishing it from prior methods focusing solely on similarity-based substitutions.

The rest of the paper is organised as follows: Sect. 2 discusses the state-of-the-art in recipe adaptation and food-related applications. Section 3 describes the methodology followed in AdaptaFood. Section 4 details the datasets. Section 5 contains the experimental setup, Sect. 6 shows the results, and Sect. 7 discusses the contributions and outcomes achieved with AdaptaFood. Finally, Sect. 8 highlights the main conclusions and future directions to pursue.

## Related work

### Food computing

Food-related studies have recently received attention from many fields, given their potential to help address population health concerns and make lifestyles healthier [5]. With the rise of social networking media, food data is being generated massively. It opens up the way to using deep learning approaches to provide automated solutions for day-to-day life. This is the context where the food computing term comes from, as the field that encompasses all those computer-based solutions with food-related data involved [1].

Food computing applications have primarily focused on tasks such as recipe and cuisine analysis [6], food recommendations [7, 8], and recipe retrieval [9]. These studies have illustrated patterns in food recipes that can be leveraged, among others, for similarity [10] and recipe generation [11]. However, these approaches rely heavily on large amounts of data and obtaining labelled food-related datasets remains a challenge [12].

Cross-modal approaches play a critical role in food computing by integrating text, image, nutrition data, and user preferences to solve specific challenges [1]. Learning cross-modal embeddings can help obtain a representation when the same object appears in multiple modalities. Modality alignment can help to align distribution from different modalities to get a joint embedding space from recipe texts and images [13–15], which is essential for tasks like automated food detection [16], recipe generation [17] and cross-modal recipe retrieval [3]. For instance, image-captioning techniques have been explored for describing food images [18] and recommending recipes based on detected ingredients [19]. However, it has not been widely applied to food tasks. One of the more promising venues is multimodal food recommendation [19]. In this study, the authors propose to recommend existing recipes based on the ingredients detected in recipe images. Image-captioning has also been used for assessing intake dietary intakes [20]. In this case, the model is used to estimate food volume.

AdaptaFood, the system presented in this work, leverages food computing advancements by integrating multimodal capabilities. Specifically, it incorporates image-captioning to allow recipe adaptation from both text and image inputs, making AdaptaFood the first approximation of multimodal recipe adaptation. It is an integral system that will enable us to modify ingredients from image and textual recipe data. As far as we know, this is the only system in the literature with the same capabilities.

### Recipe adaptation

Recipe adaptation is a relatively new research direction within food computing, with significant potential for advanced personalised nutrition and dietary support. Learning food features and relationships from recipes is crucial, as they provide insights into food combinations, flavour influence, and cultural contexts. Existing work in this area includes generating ingredient networks for recipe suggestions from recipe texts [21], understanding food pairing theory, and leveraging biochemical data for ingredient substitution [22]. For example, KitcheNette [23] applies food pairing theory to compute pairing degrees between ingredients based on recipe ingredient combinations.

Recipe adaptation is closely related to recipe recommendation tasks  [24, 25], where systems suggest recipes based on nutritional requirements [26], user preferences [27], or medical needs [28]. However, recipe adaptation goes beyond simple recommendations by modifying recipes to meet user-specific requirements. Adaptation tasks include ingredient substitution [29], text modification [30], and ingredient quantity adjustment [31]. Due to the complexity of these tasks, existing approaches typically focus on one task at a time, often using techniques like word2vec [4, 32] or ingredient networks [21]. In this study, we focus on ingredient substitution due to its potential application in many other food computing tasks, such as dietary restrictions accommodation, enhancing flavour profiles, and optimising for cost, availability, and cultural preferences of recipes.

Pre-trained language models like BERT have been successful in a wide range of language processing and understanding tasks and across different applications due to their capability for domain adaptation [33]. They allow fine-tuning for specific tasks with minimal additional data. Despite their versatility, these models may perform poorly when the target domain significantly differs from the pre-training corpus [34]. This limitation underscores the need for domain adaptation, where the target domain significantly differs from the general-purpose training corpus.

BERT-based models have recently been proposed for food-related tasks, including recipe recommendation [8], question answering in recipes [35], and ingredient quantity prediction [31]. These studies illustrate the potential of language models in understanding recipe texts and performing specific tasks. However, recipe adaptation, particularly ingredient substitution, remains underexplored in deep learning. A significant challenge is the lack of an evaluation framework to test recipe adaptations, slowing progress in this area.

AdaptaFood addresses these gaps by focusing on ingredient substitution. Unlike existing methods that rely solely on ingredient networks or word2vec models, AdaptaFood integrates external knowledge sources to add specialised nutrition information. This knowledge-based approach ensures intelligent modifications that comply with user requirements, such as dietary restrictions, flavour preferences, and cultural considerations. AdaptaFood further innovates by using SBERT to create powerful text embeddings that encode semantic relationships between food items, enabling a more nuanced and context-aware adaptation process.

Moreover, while ingredient substitution is typically performed on textual recipes, there are cases where recipe images are the only available data source. AdaptaFood bridges this gap by combining text and image modalities, using multimodal approaches to process recipes in various formats. This makes AdaptaFood the first system capable of multimodal recipe adaptation.

## Methods

Given a food recipe and a restriction set (if available), we refer to recipe adaptation as the task of obtaining new customised recipe versions from an original one. Specifically, it detects foodstuffs which are similar to the recipe ingredients in an external dataset, thus taking into account the semantics and context of the recipe. We have approached this problem in AdaptaFood from a multimodal point of view. Consequently, we deal with ingredient descriptions categorised as short texts due to their characteristics and length. Finding a suitable food substitution for a specific ingredient is a complex task, and it is difficult to tackle in a supervised manner. Food science is known for the wide variety of combinations, and factors of a very different nature can influence these relationships. For this reason, we have addressed the recipe adaptation task with an unsupervised approach. This allows us to learn features from foods while still being a feasible option to deal with unknown food combinations.

Recipe adaptation usually requires dealing with heterogeneous data sources. It is mandatory when the algorithm uses ingredient-level nutritional facts since recipe datasets do not usually include them. We have tackled this difficulty as a textual similarity problem between two datasets: the ingredient list of the recipe and a set of food items to choose from as alternatives to these ingredients. To detect items similar to the recipe ingredients, we have searched for that instance of the external set whose similarity to the ingredient description is maximum. Therefore, we calculate text similarity values between short food description texts (i.e., the recipe ingredients and the nutrient database of foods). When adapting recipes based on restrictions, we consider the similarity food values obtained with the text similarity method detailed behind, limiting the possibilities to those ingredients fulfilling the user restrictions. Indeed, it is a constrained semantic search since we seek to replace the recipe ingredients with alternative but similar foods that satisfy a specific condition.

Figure 3 illustrates the workflow of AdaptaFood, detailing the system’s input, data processing workflow, and output. The bottom section of the figure features a process line that highlights each method implemented in AdaptaFood, described as follows:

(i) **Ingredients detection:** We extract the ingredients from the input provided to AdaptaFood. When the input is a recipe image (option one in Fig. 3), we use an image-captioning model fine-tuned to the ingredient detection task. As reflected in the figure, this consists of an encoder-decoder architecture that has been fine-tuned to provide an ingredient description given an image. When the input is a recipe text (option two in Fig. 3), we get the ingredients from the recipe directly.
(ii) **Food description representation:** We build a language representation model to encode food descriptions to obtain a representation vector from the foods. It corresponds to the embedded vector resulting from averaging the word embeddings derived from the food description.
(iii) **Heterogeneous data handling:** Using the ingredient embeddings obtained with SBERT, we perform a semantic mapping to enrich the knowledge of each ingredient with information extracted from the database. To achieve this, we calculate the similarity degree between descriptions, utilizing the food representations obtained in (i) to identify equivalents and potential substitutions. This approach enables us to work seamlessly with heterogeneous data sources by detecting matching items and integrating complementary data. As shown in the corresponding step in Fig. 3, this process generates a list of ingredients enriched with additional information.
(iv) **Ingredient substitution:** We use the semantics encoded by the language model and the external sources to detect alternative foods able to substitute a specific ingredient. As a result, we obtain a list of the k-best replacements for the ingredient, as stated in Fig. 3.
(v) **Recipe adaptation:** We use the steps above to adapt a recipe by modifying its ingredients for other suitable options, thus generating customised recipe versions (see output in Fig. 3).

### Ingredients detection

AdaptaFood allows two different data inputs (recipe object or image). We extract the ingredients from the list when the input is a recipe object. When the input is an image, we use a fine-tuned image-captioning model to predict the ingredients.

The image-captioning task consists of obtaining the caption that describes a given image. Thus, it requires a computer vision model (encoder) connected to an NLP model (decoder) that learns from the visual features to generate the captions. We part from an image-captioning model published in HuggingFace [36]. This model uses ViT as an encoder, a vision transformer model re-trained on ImageNet-21k. It uses GPT-2 [37] as a decoder, a large-scale generative language model allowing higher output expressiveness. It has been trained on a sub-sample of the COCO dataset, a widely-used large-scale object detection, segmentation, and captioning dataset. We re-trained this model for the ingredient detection task, using recipe images as input and the list of ingredients as captions.

**Fig. 4:**
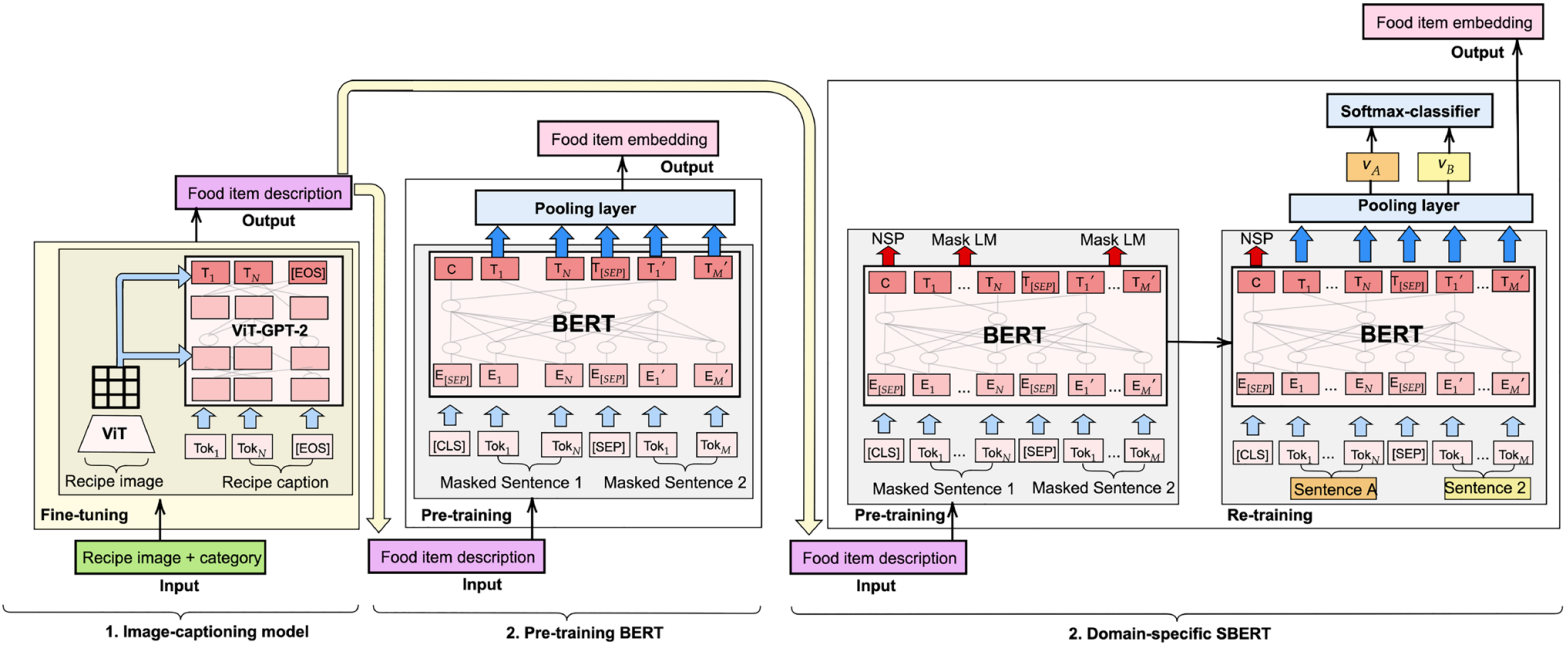
A detailed view of the image-captioning module and its integration with the BERT model architecture. The image-captioning module processes recipe images to generate descriptive captions. These captions are then fed into the BERT model, which encodes them into sentence embeddings. The setup supports both general-purpose and domain-specific BERT models

As illustrated in Fig. 4, when the input is a recipe image, the model processes both the image and its category. As an output, leveraging cross-attention with the image, the model generates a caption that describes the ingredient information of the recipe. This output is directly used as input for the language model, which encodes it as embeddings to be utilized in the subsequent steps of the AdaptaFood procedure. The figure shows two scenarios: one using a pre-trained model and the other using a domain-specific model. It is important to note that the image-captioning module operates independently of these variations.

Ingredient detection from recipe images is challenging, depending on the dish category, e.g., ingredients in a salad are more distinguishable than the ingredients of a soup or a cake [38]. For this reason, we have trained three models, one per category considered in the dataset. We have based this decision on the fact that recipes from the same type will share more properties, and it will be easier to infer the ingredients. For example, ingredient details, additional food processing included in the ingredient descriptions as well as ingredient relationships may vary depending on the category.

### Food description representation

In our previous study, we showed that non-attention-based word embedding models such as word2vec [39], GloVe [40], and Fasttext [41] perform similarly for the recipe adaptation task [4]. Attention-based models, on the other hand, may outperform those results thanks to recent breakthroughs in NLP. We propose language models based on attention mechanisms to solve the recipe adaptation problem. We have used the context-based language model BERT to build food representations from the ingredient descriptions. For this, we follow two approaches for encoding the food descriptions. We use a general-purpose BERT-based model, followed by a re-trained version on the food domain (see model architectures in Fig. 4). Specifically, we have used the SBERT architecture for building sentence representations.

**Fig. 5:**
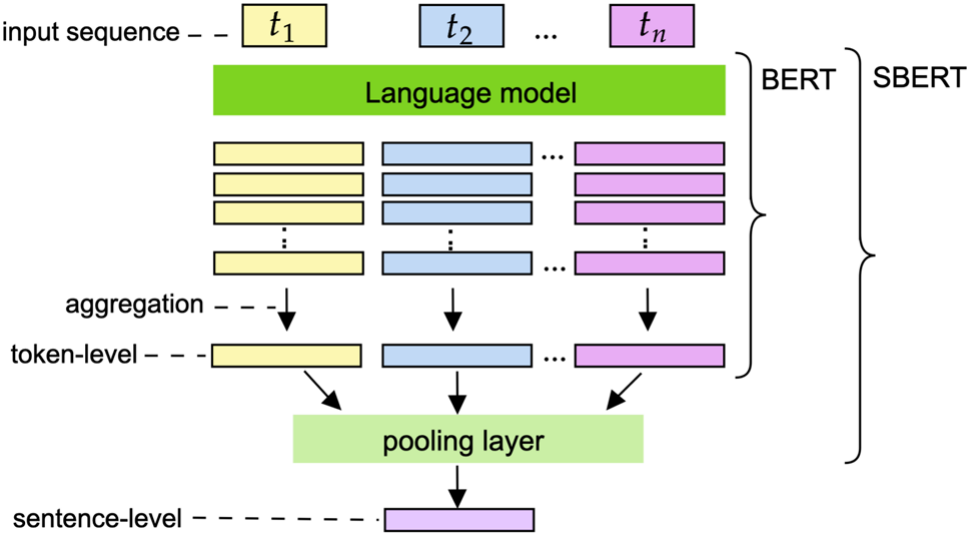
Token and sentence representation levels with BERT/SBERT

The most straightforward practice to obtain sentence vectors with BERT is averaging the token embeddings or using the CLS token instead. This approach allows us to obtain food representation vectors for ingredient substitution tasks [12]. However, these approximations can provide low-quality embeddings, sometimes even worse than non-attention-based word embedding methods [42]. Sentence-BERT (SBERT) is a BERT-based model which outperforms many state-of-the-art similarity domain-specific tasks. SBERT is a modification of BERT that uses siamese and triplet networks to generate semantically meaningful sentence-level embeddings [42]. SBERT directly returns a sentence representation corresponding to the input sentence [42]. A pooling operation (e.g., mean vector or max-over-time) allows the building of a single representation from the token embeddings obtained with BERT. Thus, we distinguish two levels of representation of the data (see Fig. 5):

1. Token-level representation: BERT generates a representation vector per each token in the input sequence. For each token, this vector aggregates the data embedded in the hidden layers of the network. Consequently, a decision must be made on which hidden layers to consider in the final token representation vector.
2. Sentence-level representation: SBERT aggregates token-level representations (i.e., the last layer of the last hidden state of BERT) to build a unique sentence representation vector. Which aggregation method is most suitable for obtaining the sentence vector has to be decided.

In Sect. 5, we evaluate the model at both levels to decide the combination of the layers for the token-level vectors and the aggregation strategy for the sentence-level vectors.

#### General-purpose language models

We use BERT, a general-purpose pre-trained language model to encode the food descriptions [33]. Food descriptions are usually sentences. We are able to obtain their encoded representations by aggregating their word vectors. BERT provides a 768-dimensional embedded vector for each token in the input sentence, allowing us to extrapolate tokens and get a sentence representation.

#### Domain-specific language models

General-purpose BERT is fully trained on Wikipedia, but the nature of our data is slightly different since recipe data has a specific scope and style of writing. Food recipes are characterised by several short sentences containing ingredient instructions and imperatives. It contains common verbs that tend to appear in many recipes (e.g., boil, cook, fry, etc.). A similar course of action also exists since many ingredients are common and preparation styles and food combinations may overlap. Adapting BERT to the food domain allows us to learn food features and enrich vector representations with knowledge-specific data.

**Re-training to the food domain.** We have used a food-related corpus containing recipe text instructions for preparing the dish (see detailed data source in Sect. 4). These sentences can help us to learn from context related to how ingredients are prepared, cooked, and combined to make a tasty recipe. We decided to re-train BERT for the next sentence prediction task (NSP). This task fits with the training dataset as we are dealing with preparation texts, where the order of the sentences is relevant. It means that each sentence corresponds to a recipe step, and the recipe steps are described in a recipe in a sequenced manner. Figure 4 shows the process of re-training the model. It receives a pair of sentences as input. The output indicates if they are consecutive or not. The experimental section (Sect. 5) provides details about the re-trained model, the selection of hyperparameters, the evaluation criteria, and the experimental setup required to obtain the domain-specific language model.

**Fig. 6:**
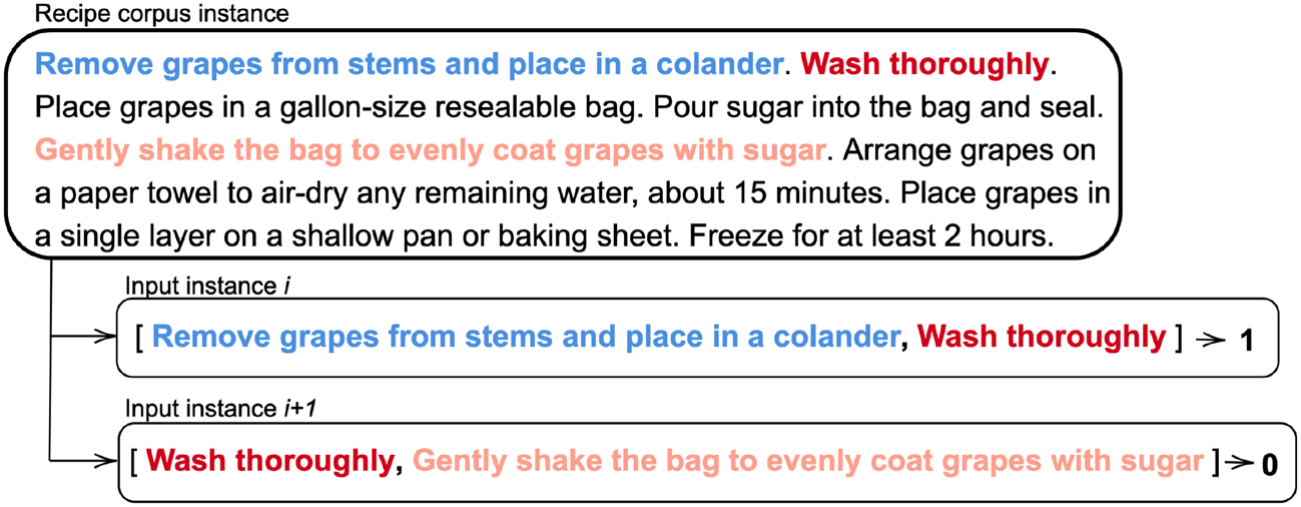
Procedure to build the training set

**Building the training dataset.** We have built the training corpus from scratch using the text instructions from the recipe corpus. We randomly generate pairs of sentences, and a label indicates whether the sentences are consecutive (see Fig. 6). It shows an example of how we generate the training pairs from a sample of the recipe corpus. In this example, the pair [“*Remove grapes from stems and place in a colander*”, “*Wash thoroughly*”] corresponds with the two following sentences, and therefore, the assigned label is 1. Otherwise, the pair [“*Remove grapes from stems and place in a colander*”, “*Gently shake the bag to evenly coat grapes with sugar*]” the first sentence is not followed by the second one in the original text and the label is 0 for this case. Section 4 includes the details of the dataset used for creating the ingredient corpus.

### Heterogeneous data handling

Sentence-based models and similarity measures such as cosine similarity or Euclidean distance allow the finding of semantically comparable sentences [42]. In our case, we have used the cosine similarity distance. We found equivalent items to the recipe ingredients in the food composition table to expand the nutrition information for the ingredients. We used the sentence embedding of the food items (i.e., ingredients and food composition table items) to calculate the similarity degree between their embedded vectors. This allows us to map the ingredients to the external food dataset.

Regarding diversity in nutrition data, we would like to emphasize that we are using nutrition resources maintained and validated by EuroFIR,^5^ a non-profit organization that provides validated worldwide food composition databases. This extensive initiative ensures that the databases we rely on are validated, offering consistent alignment between nutrients, standardized units, and reliable information across European nutrition databases. We chose to work within this framework because it guarantees the robustness and validity of the nutritional data, which is maintained and endorsed by reputable health associations. Additionally, its content is consistent and easy to integrate with standard global nutrition databases, ensuring seamless interoperability.

Regarding diversity in ingredient data, we acknowledge the challenges of managing ingredient diversity across various sources. As highlighted in [4], ingredient diversity primarily stems from varying levels of detail, as ingredient descriptions in recipes are often less specific compared to those in specialized nutritional resources. For cases where no optimal adaptation exists for an ingredient, we employ a compatibility score with a threshold in the range (0, 1). This score is defined based on the nutritional deviation and cosine similarity of potential substitutions, with the threshold adjusted according to the specific dietary restriction. If no substitution meets the threshold, no substitutions are provided. Currently, this method is implemented for non-restricted, vegan, and vegetarian options. Sect. 6.5 presents user satisfaction metrics for the substitutions, which provide evidence supporting the effectiveness of our approach. Additionally, we have integrated an evaluation of the compatibility score into the human annotation platform to assess its interpretability and usability for end-users.

### Ingredient substitution

Both the semantics included in the ingredients encoding and ingredient features aggregated from the specialised external dataset allow us to filter the ingredients and suggest food options to build the adapted recipe and comply with the user’s requirements. We use the food embeddings and the cosine similarity to find the best substitution choices in the external dataset.

In CoFID, the manual provides clear guidelines on interpreting and managing missing values. These are indicated as Tr (trace amounts of a specific nutrient) or N (a significantly high amount of the nutrient, but not quantified). Our approach substitutes Tr values with 0.0001 and N values with the median of that nutrient within the corresponding food group-a commonly adopted solution in nutrition studies.

It is important to emphasize that this approach does not compromise the validity of our methodology. Macronutrients, which are the primary focus of our adaptations at this stage, are comprehensively detailed in CoFID. However, the missing values primarily relate to minerals and other micronutrients, which are highly relevant but represent hyper-specialized areas of recipe adaptation. Addressing these areas requires much more specific and detailed data, and as such, they are not currently covered by our model. Expanding into these high-specific domains would involve considerations more aligned with health and disease treatments, which are beyond the present scope of AdaptaFood.

Regarding missing nutrients, we identified a specific issue with CoFID, as it does not provide information on salt content. To address this, we inferred salt content from sodium values and included it as a nutrient in our resource. This decision reflects our belief that software designed for public use should present information in a way that is easily understandable. Salt, being more intuitive and familiar to non-expert users than sodium, enhances accessibility for the general population.

### Recipe adaptation

With the methods described above, the workflow of AdaptaFood results as shown in Fig. 3. The method receives as input three different objects as input: (1) a recipe with a list of its ingredients, (2) an external food dataset with nutrition facts of several common ingredients, and (3) a list of user requirements for filtering food external datasets according to the user’s needs. We preprocess the food descriptions from inputs (1) and (2) to obtain corresponding embeddings with the language model. Finally, (1) and (2) food descriptions are aligned using their embeddings to detect if they meet user requirements. Otherwise, the method suggests similar (and allowed) substitute options from the external food dataset. Both the alignment and the ingredient suggestions are obtained by doing similarity/semantic tasks towards their embeddings.

Regarding the data integration task, the inputs involved are two: (1) the recipe to adapt and (2) the complementary data we want to include in the adaptation process (i.e., dietary and nutrition-specialised data at the ingredient level). For the adaptation task, the data involved are the recipe, the already integrated dataset, and the user’s requirements (if provided).

We would like to remark the rationale behind our approach. As stated before, we combine well-known architectures in NLP and computer vision, such as BERT, ViT, and GPT. We hypothesize on integrating these methods to address the ingredient substitution problem and explore the benefits of retraining in the food domain.

Regarding the ViT-GPT architecture for image captioning, several previous works have affirmed its suitability for the task [43, 44]. The generative textual capabilities of GPT, combined with cross-attention mechanisms in the ViT transformer, enable the model to generate contextual descriptions based on images.

For the BERT-based architecture for ingredient substitution, the use of pre-trained language models has been highlighted in state-of-the-art ingredient substitution approaches [12]. Since recipe data is structured at the sentence level, we believe that elevating the abstraction of textual representation to the sentence level using Sentence-BERT models is crucial for optimizing this task in the food domain, as it effectively captures sentence-level semantic relationships between the ingredients and cooking processes. As stated later, for the re-training to the food domain in recipe texts, we address the problem like a similarity problem between food descriptions. In this sense, we re-train the model using NSP, assuming that two sentences are more similar if they appear one beside the other in the food descriptions than if they are chosen randomly. We must highlight that this assumption is the same used in [45] for the same task.

## Data

This study has required data from different sources to tackle the many methods involved in the adaptation task. We have used the *MealRec* dataset for fine-tuning an image-captioning model for the ingredient detection task. For re-training the language model to the food computing field, we have used the *recipe instructions training corpus*. We have used the *CoFID dataset* and the *recipe ingredient corpus* to test the ingredient substitution algorithm. It allows us to evaluate the quality of the text representations powered by the language model. We have used the *Food.com recipe collection* and the datasets used for the ingredient substitution to test the adaptation method. Below we detail the data involved in each step of the experimentation.

**MealRec.** We have used MealRec [46], a meal recommendation dataset, to fine-tune an image-captioning model to detect ingredients from images. It contains 7,280 recipes from three categories (appetiser, main dish, and dessert). Each recipe includes the image and the ingredients, among others.

**Recipe instructions training corpus.** For re-training BERT, we have used a recipe corpus collected from many food sources available online used in [4]. It contains 267,071 recipes from *AllRecipes*, *Epicurious* and *Cookstr*.

**Recipe ingredients corpus.** We have built an ingredient corpus to have a representative set of foodstuffs. We have used five recipe collections to extract the ingredient list. Some of these resources contained typographical errors and misspellings, as they belong to online blogs managed by user communities. We have developed a homogenisation of the text, removing symbols, punctuation marks, and non-alphanumeric characters. We have also ignored the content in brackets since it is additional but not essential information, and we have singularized the texts. After combining these sources, we obtained 104973 ingredients (9800 unique values). We used the following data sources: (1) a scraped collection of BBC Foods,^6^ (2) the ingredient collection used in the BBC Recipes dataset, (3) the *Recipe ingredients Dataset* from a Kaggle competition,^7^ (4) the *Indian Food 101* dataset with Indian recipes (also available at Kaggle^8^), and (5) the *Foods Recipes* dataset from Archana’s Kitchen also available at Kaggle.^9^ In this way, we have ensured that the recipes used for the ingredient corpus differ from those used for the model training.

**Composition of the foods integrated dataset (CoFID).** We have used the CoFID dataset^10^ to complement specialised ingredient information. It is maintained by the Public Health Agency (PHE) of the Department of Health and Social Care in England. We have used the *Proximates* table for our task since it contains nutrition information on 2913 up-to-date food items, thus allowing for extending recipe ingredients data to consider nutrition facts. For the food item descriptions, we have applied the same cleaning procedure described for the recipe ingredient corpus. We excluded the items related to cooked dishes and human milk items to avoid using them in our approach (our approach suggests food ingredients and not recipes). We have also used two additional columns to indicate if the food is vegetarian and/or vegan, which we preprocessed in a previous study [4]. We call this dataset “external food dataset” since it provides external knowledge to AdaptaFood.

**Food.com recipe collection.** We have tested AdaptaFood with recipes obtained from the Food.com recipe collection,^11^ which has been used before for many food computing applications [47, 48]. It contains, amongst many others, recipe data regarding ingredients, recipe instructions, and titles.

## Experiments

AdaptaFood is a complex system that conducts several tasks for the recipe adaptation goal. Below, we describe all the steps to obtain a successful adaptation. AdaptaFood is multi-modal (it accepts images or recipe text to perform the task). For this reason, the first step (i.e., image-captioning) can be omitted if the input is a recipe text that includes the ingredient list.

### Action line

1. Firstly, we fine-tune the image-captioning model detailed in Sec. 3.1 with the MealRec dataset for the three categories. We analyse their performance for detecting ingredients from the given image and with an additional validation in Food-101. We also conduct a category-guided analysis to study the model performance across the recipe categories of MealRec. This allows us to obtain the food descriptions of the ingredients when the provided input is a recipe image.
2. Once we have the ingredient list, we study the food representation embeddings obtained at token-level representation. Specifically, we focus on selecting the last hidden state layers for building the token vector. We use BERT for this experimentation and select four strategies commonly used for token representation vectors: (i) the average of all layers, (ii) the last layer, (ii) the penultimate layer, and (iv) the concatenation of the last four layers. We also compare these results with SBERT (which uses the last layer state of the tokens to build the sentence embedding) due to the high-quality results for sentence-based applications in the literature. All these sentence-level embedding strategies aggregate the token vectors of the sentence using a mean pooling operation.
3. We study the sentence-level representations obtained with SBERT following three strategies for token aggregation: (i) the mean of token vectors, (ii) max pooling operation when the sentence embedding corresponds to the maximum token vector of the sequence, and (iii) the CLS token embedding to represent the sentence.
4. We re-train the model to the food computing domain. We use the best token and sentence-level representations from steps (2) and (3).
5. We analyse the results, focusing on general-purpose and specific-domain models in the recipe-adaptation task. We also compare the performance of SBERT pre-trained and domain-specific models with the word2vec approach we proposed in [4] for the recipe adaptation task. From here on, we refer to this model as “Domain-specific word2vec”. For this, we conduct human, nutritional, and ground-truth evaluation.

### Experimentation setup

First, we describe the image captioning model used for extracting the ingredients if the input is an image. Then, we detail the pre-trained BERT model and how we re-train the model for the food computing domain. Finally, we detail our application task in recipe adaptation.

#### Image-captioning model

We parted from a pre-trained ViT-GPT2 model for image-captioning, a common approach in previous studies on image captioning [43, 44]. The encoder is based on ViT [49]. The resolution of the input image is 224x224, and that model splits into patches of 16x16 pixels. It consists of 12 transformer layers, 12 attention heads, a hidden size of 768, and an intermediate size of 3072. The decoder is based on GPT, with GELU new as activation function, a dropout rate of 0.1, and cross-attention to also consider the image in the predictions. It consists of 12 attention heads for each of the 12 hidden layers. The dimension of each hidden layer is 768. The number of tokens in the vocabulary is 50,257, and the maximum context size is 1024.

For the fine-tuning, we trained each model during three epochs, which was selected by the one that achieved by the best loss in validation. The batch size is 4, and the learning rate is $5\cdot 10^{-5}$. We have decided these hyper-parameters after studying the over-fitting of the models. We have used BERTScore [50] as the primary evaluation metric to calculate accuracy, recall, and F1. BERTScore is an automatic evaluation metric for text generation that compares each token in the predicted sentence to each token in the target sentence, determining their similarity based on cosine similarity. We have used it with the aim of improving alignment between image features and generated captions. To complement these results and provide more interpretability, we have also incorporated SemScore [51] as an additional metric. SemScore captures the semantic alignment between generated captions and ground-truth descriptions, particularly emphasizing context-specific details. This makes it especially well-suited for image captioning, as it evaluates whether the predicted caption aligns with the image’s content, even if the phrasing differs from the reference captions. The inclusion of SemScore has added finer granularity to our analysis, enhancing our results’ interpretability.

We have applied the same process across the three recipe categories: appetisers, main dishes, and desserts. After excluding recipes with missing values, we have obtained a total of 2,735 appetiser recipes, 2,552 main dishes recipes, and 1,991 dessert recipes. We have split each subset into 80/10/10 proportions for training, validation, and testing, respectively.

To extensively evaluate the image-captioning method, we have trained the model on an additional dataset, Food-101. This external validation allowed us to assess the model’s performance on larger datasets and identify potential limitations.

Food-101 [52] is a widely-used food recognition dataset comprising 101 food categories. This large dataset presents diverse food dishes, varying in difficulty, origin, and cultural context. The images within the dataset exhibit significant variance in food type, colour, exposure, and level of detail. Additionally, the dataset includes visually and semantically similar food types, posing a challenging task for recognition models. This diversity makes Food-101 particularly interesting for our analysis. While direct comparison with Meal2Rec is limited due to the absence of complete ingredient lists, Food-101’s focus on simple dishes provides a unique dataset for exploring the capabilities of the image-captioning model. This simplified task allows us to assess how well these models can identify and describe essential ingredients and recipes.

To improve the performance on the evaluation metrics, the batch size was adjusted to 32. This dataset includes predefined training and testing sets; however, we further split the training set to create a validation set for evaluation during training. Consequently, the training, validation, and test sets contain 60,598, 15,149, and 25,250 samples, respectively. We followed the same approach as in MealRec, training a single model since category information was not provided. The training and evaluation methodologies, as well as the encoding of both image and text, were kept consistent. The model was trained for up to 15 epochs with early stopping, achieving the best results at epoch 5, which was selected as the final model.

#### General-purpose language models

Deciding if a specific food item is a suitable replacement for an ingredient in a recipe is subjective. It depends on many factors, such as the rest of the ingredients, cooking procedures, user preferences or cultural factors and traditions. Consequently, there could be more than one valid solution for a food replacement [4, 12]. Consequently, there is a lack of ground truth datasets to evaluate our method with. To evaluate the method quantitatively, we compare how many ingredients are captured. Note that this is a lower performance bound due to the fact that similar ingredients may have different spelling in specific situations. Then, in the following experiments, we will evaluate the best methods qualitatively in our real-world task of recipe adaptation. This is helpful for the food computing heterogeneous data alignment task due to the high probability that the equivalent food items share syntactic properties. We use the Jaccard score for measuring syntactic similarity. The lower bound value lies between 0 and 1. The 0 denotes no similarity between the two food descriptions, and 1 denotes complete syntactic matching.

We have used the 12-hidden layers version pre-trained BERT model available in HuggingFace^12^ with the architecture and parameter configuration detailed in [33]. For the sentence model, we have used the pre-trained sentence-transformers model (SBERT), with both the architecture and parameter configuration from [42].

#### Domain-specific language models

We re-trained the model for the next sentence prediction (NSP) task to detect if one sentence follows the original one in the recipe. We pursued two strategies to re-train the model:

(i) We first implemented a classification model using soft-max and cross-entropy as loss function. We tested the results by evaluating the accuracy values achieved with the model when labelling the test subset.
(ii) We used the contrastive loss as a loss function (1 if the distance between the sentences is lower and 0 for the opposite). This strategy lies in the fact that there is a greater likeness and consistency between two sequential sentences in a recipe than if the sentences were chosen randomly. This brings us closer to a problem of similarity between food descriptions, which is our ultimate goal. We have evaluated the model based on the similarity of the embeddings using the Manhattan distance, the Euclidean distance, and the cosine similarity.

We implemented the model using the functionalities of the SentenceTransformers framework  [42]. We obtained a balanced subset of 12,000 samples (6,000 per class) from the recipe instructions corpus. 10,000 of them were used for training and the remaining for testing. We conducted a homogenisation of the text, removing symbols, punctuation marks, and non-alphanumeric characters, which are not relevant for our task. For text tokenization, we follow the approach required by SBERT, which relies on the WordPiece technique. This technique splits words into smaller subwords found in the SBERT vocabulary. Additionally, a special token [CLS] is added at the beginning of each sentence to serve as a summary representation of the sentence. We use a fixed sequence length of 128 tokens. For sentences shorter than 128 tokens, we add PAD tokens to ensure a consistent length. For longer sentences, we truncate the text to fit within the 128-token limit.

We re-trained the SBERT model over four epochs, employing the two training strategies detailed in Sect. 5.2.3. The SBERT architecture builds upon a pre-trained BERT model as its backbone and integrates a Siamese network structure. This design enables the detection of sentence similarity by processing two sentences independently through identical BERT models. In our re-training process, the SBERT architecture learns to capture the semantic relationships specific to the recipe sentences in our training corpus. By leveraging the pre-trained backbone and adapting the model to our domain, we enhanced its ability to discern food-based semantic similarities and differences in the context of culinary recipes. For the first strategy (i.e., soft-max and cross-entropy), we trained the model during 4 epochs with regular evaluations every 100 steps. The model reached the best results by setting the batch size to 16, the learning rate to $2\cdot 10^{-8}$, and warm-up steps to 1,500. We obtained an accuracy value of 0.5928 and 0.6034 in the training and test sets, respectively. For the second strategy (i.e., by using contrastive loss), we set the batch size to 16, the learning rate to $2\cdot 10^{-7}$, and warm-up steps to 3,000. We re-trained the model during 4 epochs. We obtained accuracy values of 0.624 and 0.625 for training and test, respectively. The hyper-parameters were chosen after the experimentation based on the above evaluation metrics.

#### Recipe adaptation

Among all the detailed strategies for obtaining sentence representations described above, we have used the one that has given us the best results during the experimentation (i.e., domain-specific SBERT using max pooling). To provide adaptations to the given ingredients, we implemented the option of including restrictions on the food items returned by our algorithm. In particular, we have considered restrictions for vegetarian and vegan diets. We propose them as an example of how to use AdaptaFood to adapt a recipe to plant-based diets, promoted for healthy and balanced diets [53].

We have evaluated AdaptaFood in three stages. First, we have conducted human annotation to better assess the quality of results across different models. Since the task is highly subjective, human annotation serves as a reliable resource for evaluating the quality of adaptations [4, 12]. From the nutritional perspective, we have examined the nutritional variations that provide the substitutions over the original recipes. Additionally, we have analysed the adaptations from the perspective of food groups and their persistence throughout the adaptation. The experimentation has also studied the three types of adaptations to understand how the model behaves depending on different restrictions. Finally, we have developed an evaluation over ground-truth evaluation sets to analyse the quality of the adaptations, with the aim of obtaining more quantitative results over the substitutions in a broader scope. Since the ground truth lacks information about food restrictions, we have calculated evaluation metrics-Accuracy, Recall, and F1-score-using the model without restrictions.

***Human evaluation***

We have developed a human annotation platform that allows users to evaluate the quality of adapted recipes and assess our methodology regarding user satisfaction through a real-world experiment. This platform provides users with adapted recipes generated by the models tested in this study: (1) the general-purpose Sentence-BERT, (2) the food-domain-specific retrained Sentence-BERT, and (3) a previous model based on Word2Vec [4]. The recipes have been adapted to three modalities: unrestricted adaptation, vegetarian adaptation, and vegan adaptation. Once users access the annotation platform, they annotate 15 recipes from various adaptation types and models, which are presented randomly while ensuring an equitable distribution across annotators.

In the platform, users are presented with the recipe title, original ingredients, and preparation steps to provide context for the adaptation. Each adaptation is evaluated by the same user at two levels:

1. **Ingredient level**: For each ingredient, we present the top-5 best replacements identified by the model. Users are provided with an ingredient compatibility score alongside each replacement, quantifying the similarity between the original ingredient and its replacement. This is intended to offer users as much interpretable information as possible. Users are then asked to individually rate each replacement on a scale of 1 to 5, where 1 indicates a poor evaluation, and 5 is an excellent one. As a result, we collect 1-to-5 ratings for each of the five proposed replacements for every ingredient in the recipe.
2. **Recipe level**: Users are asked to answer their overall satisfaction with the adaptation and the compatibility score of the substitutions. Each question is rated on a 1-to-5 scale as well.

***Nutritional evaluation***

We have studied the replacements proposed by our approach from a nutritional perspective. From all the evaluated adapted recipes, we extracted the 5-top best replacements suggested by the model. We analysed the nutritional information of the adaptations by calculating the increase introduced by the new ingredient replacements regarding the original one. However, this increase is not normalized because the significance of the change depends on the nutrient itself. For instance, a 10 g increase in sugar represents a substantial difference, while the same 10 g increase in carbohydrates might be considered less impactful. Therefore, the interpretation of the increment varies based on the nutrient being analysed. In order to provide more interpretation here, we have also considered the reference daily value of each nutrient following the FDA. Given the ingredient *i* and the replacement *k*, we calculate the normalised increment for the ingredient *n* as it follows:

1 $$\begin{aligned} \text {Normalized Increment}_{n} = \frac{|\text {Value}_{k,n} - \text {Value}_{i,n} |}{\text {Reference}_{n}} \end{aligned}$$

For this study, we have included energy (kcal), protein, carbohydrates, sugar, fat, fibre, and salt to represent the recipes’ nutritional profiles comprehensively. We analyse the recipes without restrictions for these nutrients. On the other hand, vegetarian and vegan adaptations often require replacing ingredients with substantially different nutritional profiles, making it less meaningful to analyse variation in these cases, as significant differences are inherent to the adaptation process. Therefore, to better understand the behaviour of our model under dietary restrictions, we have primarily analysed these adaptations separately.

***Ground-truth evaluation***

To evaluate the performance of the models in ingredient substitution, we employed the ground-truth dataset introduced in [12], which contains 13,056 pairs of food ingredients and their corresponding substitutions. To the best of our knowledge, this dataset represents the most comprehensive benchmark for ingredient substitution, encompassing a wide variety of recipes.

The primary goal of AdaptaFood is to create recipe adaptations tailored to specific dietary needs. In this context, ingredient substitutions must meet strict criteria to be deemed valid, emphasizing the importance of the context. AdaptaFood relies on external food resources to propose adaptations that fulfill these criteria, meaning the proposed ingredients must originate from these sources. This dependency can complicate direct comparisons with other methods, as, to the best of our knowledge, no ground-truth datasets exist to identify the most suitable substitutions from a given set of options. Instead, existing resources generally provide collections of ingredient-substitution pairs. To address this limitation, we have restructured our experiments using a ground-truth dataset with two primary objectives:

**Study 1: Quality of our substitution approach.** The first objective is to evaluate whether, in the case of a ground-truth substitution, our model can accurately predict it. To achieve this, we leverage the embeddings and compatibility scores from AdaptaFood. We calculate Recall, MSE, and MAE, considering that the model should assign a compatibility score of 1 to the substitute proposed in the dataset. This allows us to assess how well the embeddings align with the ground truth.

**Study 2: Evaluation of ingredient substitutions.** The second objective focuses on evaluating ingredient substitutions. Since the dataset contains ingredient-substitution pairs, with multiple substitutions available for the same ingredient, we calculate Accuracy, Recall, and F1 scores, based on how many, from those substitutions, we could reach with AdaptaFood. We averaging the results at the ingredient level to obtain general accuracy, recall and F1. Notably, as guided by the compatibility score, the Recall metric provides valuable insights for both studies 1 and 2.

We would like to highlight that the ground truth allows for evaluating substitutions that are indeed correct. However, ingredient substitution is an open-ended task with many possible valid options. Consequently, the fact that the model suggests a substitute not included in the ground truth does not necessarily mean it is incorrect.

## Results

### Image-captioning for ingredient detection

**Fig. 7:**
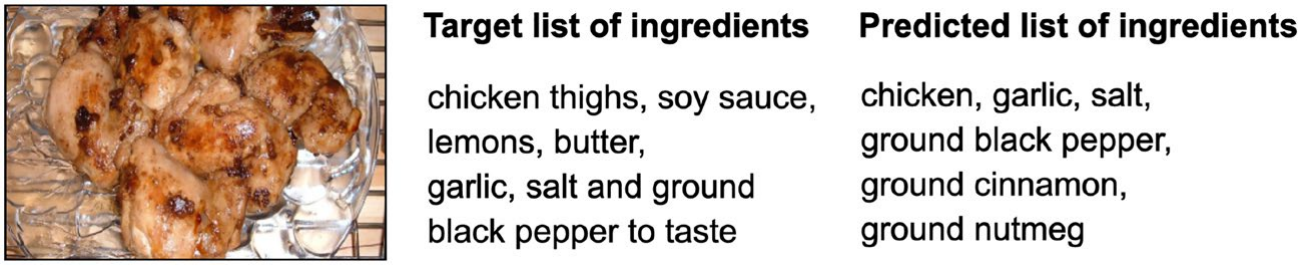
Differences in the detail and indistinguishable ingredients with the image-captioning model

Table 1 shows the precision, recall, and F1 obtained with the validation subset. Each row corresponds to the model trained in one of the categories. Figure 7 shows an example of the captions generated by the model.

**Table 1:** Image-captioning model performance over the datasets

Dataset - Category	Precision	Recall	F1	SemScore
MealRec - Appetiser	0.8858	0.8396	0.8618	0.6858
MealRec - Main dish	0.8819	0.8395	0.8600	0.6827
MealRec - Dessert	0.9011	0.8604	0.8801	0.7408
Food101 - (no categories)	0.856	0.8963	0.8751	0.7467

**Fig. 8:**
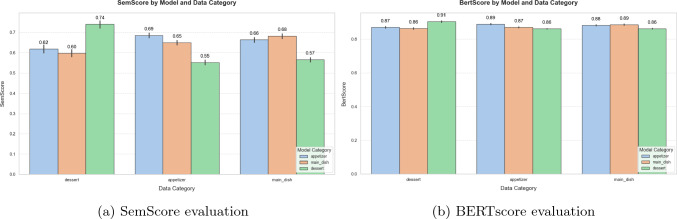
Comparison of similarity scores by image-caption model and data category

Figure 8a and b reveal significant cross-model differences in the dessert category. Models fine-tuned on dessert recipes consistently achieved superior results within this subset, effectively capturing its unique characteristics. In contrast, models trained on appetisers or main dishes performed poorly on dessert recipes, highlighting limited generalisation across categories. While the performance gap between appetisers and main dishes was modest, desserts stood out as a distinct and challenging category.

**Fig. 9:**
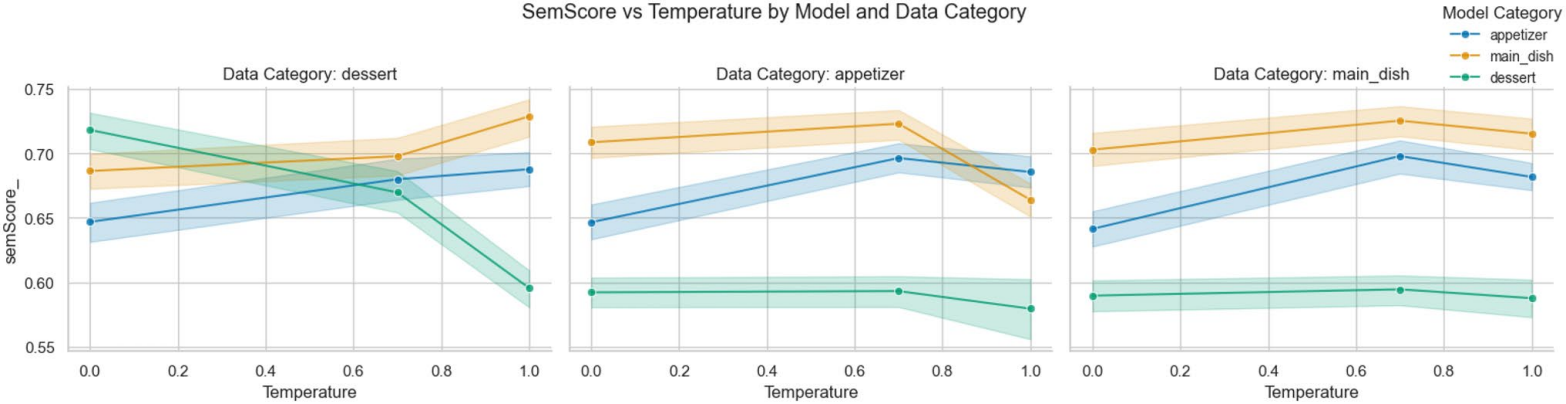
Variation of SemScore for different temperatures

Temperature variations further revealed nuanced model behaviour. As shown in Fig. 9, lower temperatures resulted in poor performance for desserts. In contrast, higher temperatures, which introduce more significant variability, sometimes improved results for appetisers and main dishes. This suggests that performance in certain categories may benefit from controlled variability in model outputs.

### Token-level representation

Table 2 shows the results obtained with the lower bound described in Sect. 5.2 for the four token-level strategies. Each row comprises the values obtained with the lower bound for a specific strategy. The first column (i.e., “representation strategy”) contains the subject strategy, and the “top *k*” columns contain the lower bound value for the *k*-best mapping reached in the external food dataset. Note that this experimentation has been applied to 100 ingredients, with 100 being the maximum lower bound value.

**Table 2:** Lower bound values for the token-level representation strategies (sentence embeddings obtained using mean pooling)

Strategy	Top 1	Top 2	Top 3	Top 4	Top 5
BERT - Avg. all layers	**55**.**16**	**30**.**50**	21.16	**21**.**33**	14.66
BERT - Last layer	36.66	11.50	6.83	9.50	6.66
BERT - 2nd last layer	38.66	13.00	11.66	8.00	8.66
BERT - Concat. 4 last layers	38.66	15.50	11.50	10.33	6.66
SBERT - Last layer	48.16	27.83	**26**.**16**	18.50	**16**.**33**

### Sentence-level representation

Table 3 shares the same structure as Table 2 but compares sentence-level representations with SBERT. The rows contain the lower-bound values achieved with each strategy used for sentence-level representation (token-level embeddings in SBERT are represented with the last layer state). The first and second rows contain the values using the mean and max pooling of token levels when building the sentence, respectively. The third row comprises the lower-bound values when using the CLS token embedding to represent the sentence.

**Table 3:** Lower bound values obtained for the three chosen sentence-level representation strategies with SBERT

Strategy	Top 1	Top 2	Top 3	Top 4	Top 5
Mean pooling	48.16	27.83	**26**.**16**	18.50	16.33
Max pooling	50.66	**31**.**66**	23.16	**22**.**83**	**21**.**83**
CLS	**53**.**16**	27.16	24.83	21.16	20.83

### Domain-specific BERT

Table 4 compares the results obtained with the general-purpose SBERT and the domain-specific SBERT following the second strategy. It includes the results for the domain-specific word2vec model. It shares the same structure as Tables 2 and 3. The first row corresponds to the general-purpose model, which shows the best behaviour (i.e., last layer embedding and max pooling for token and sentence level representation, respectively). The second row shows the results after re-training SBERT in the food domain. The third row contains the results for the domain-specific word2vec model.

**Table 4:** Lower bound results for the SBERT pre-trained model and the re-trained SBERT model to the food computing domain

Model	Top 1	Top 2	Top 3	Top 4	Top 5
Pre-training SBERT	50.66	31.66	23.16	22.83	**21**.**83**
Domain-specific SBERT	**57**.**00**	**37**.**33**	**31**.**16**	**23**.**33**	21.00
Domain-specific word2vec	30.33	27.16	22.50	19.66	18.00

### Recipe adaptation

Once the model was validated, we used it to generate adapted recipe versions. We obtained the five most accurate replacements for each ingredient in a recipe based on the semantics captured by the language model. Tables 5 and 6 show the results obtained with the general-purpose and domain-specific models in Table 4, respectively. Both tables share the same structure. The first column represents the k-best returned ingredient, and the remaining columns correspond to ingredients in the recipe. The first column represents the *k*-best position, whilst the other columns refer to the recipe ingredients to obtain food substitutions. Tables 8 and 9 show examples of adaptations for vegetarian and vegan diets using the domain-specific SBERT model.

**Table 5:** An example of the food replacements suggested by AdaptaFood for adapting a recipe using the general-purpose SBERT model (no dietary requirements considered)

k	Cheddar cheese	Butter	Sour cream	Green onions
1	Cheese, Cheddar, English	Butter, spreadable	Dips sour, cream based, assorted flavour	Cabbage, green, raw
2	Cheese, Edam	Ghee butter	Dips sour, cream based, reduced fat	Beans, green, raw
3	Cheese, Ricottum	Butter, spreadable, light	Vinegar	Broccoli, green, raw
4	Cheese, Camembert	Butter, salted	Toffees, mixed	Peppers, capsicum, green, raw
5	Cheese, Quark	Cocoa butter	Raspberries, raw	Broccoli, green, steamed

**Table 6:** An example of the food replacements suggested by AdaptaFood for adapting a recipe using the domain-specific SBERT model (no dietary requirements considered)

k	Cheddar cheese	Butter	Sour cream	Green onions
1	Cheese, Cheddar, English	Butter, spreadable (75–80% fat)	Dips sour cream based, assorted flavours	Peppers, capsicum, green, raw
2	Cheese, Cheddar type, half fat	Butter, salted	Dips, sour-cream based, reduced fat	Cabbage, green, raw
3	Cheese, Edam	Butter, spreadable, light (60% fat)	Cream substitute, single	Plantain, green flesh, raw
4	Cheese, Ricotta	Ghee, butter	Cream substitute, double	Beans, green, raw
5	Cheese, Camembert	Butter, unsalted	Sugar, icing	Green beans, dried

**Table 7:** An example of the food replacements suggested by the recipe adaptation method we implemented in [4] for adapting a recipe with no dietary requirements considered

k	Cheddar cheese	Butter	Sour cream	Green onions
1	Cheese, Cheddar, English	Butter, unsalted	Dips, sour-cream based, assorted flavours	Onions, raw
2	Cheese, Derby	Ghee, butter	Dips, sour-cream based, reduced fat	Grapes, green
3	Cheese, Caerphilly	Cocoa butter	Bran, wheat	Pesto, green
4	Cheese, Cheddar, 30% less fat	Butter, salted	Piccalilli	Peppers, capsicum, green, raw
5	Cheese, Cheddar type, half fat	Butter, spreadable (75-80% fat)	Lager, alcohol-free	Onions, baked

**Table 8:** An example of the food replacements suggested by AdaptaFood for adapting a recipe following vegetarian dietary requirements

k	Condensed tomato soup	Bacon	Spaguetti noodles
1	Soup, cream of tomato, canned	Cheese, Paneer	Squash, spaghetti, baked
2	Tomato juice	Cheese, Edam	Pasta, spaghetti, canned, in tomato sauce
3	Soup, tomato, carton, chilled	Courgette, fried in butter	Squash, spaghetti, raw
4	Tomato ketchup	Cheese, Ricotta	Pasta, wholewheat, spaghetti, dried, raw
5	Baked beans, canned in tomato sauce	Cheese, Quark	Pasta, white, spaghetti, dried, boiled in unsalted water

**Table 9:** An example of the food replacements suggested by AdaptaFood for adapting a recipe following vegan dietary restrictions

k	Condensed tomato soup	Bacon	Spaguetti noodles
1	Soup, cream of tomato, canned	Pizza base, raw	Squash, spaghetti, baked
2	Tomato juice	Bread, ciabatta	Pasta, spaghetti, canned, in tomato sauce
3	Soup, tomato, carton, chilled	Bread, pitta, white	Squash, spaghetti, raw
4	Tomato ketchup	Currant buns, toasted	Pasta, wholewheat, spaghetti, dried, raw
5	Baked beans, canned in tomato sauce	Bread, white, ‘with added fibre’	Pasta, white, spaghetti, dried, boiled in unsalted water

***Human evaluation***

For the user satisfaction study, we adapted 66 recipes from the Food.com dataset to the three considered adaptation types. These recipes were evaluated by 20 users, from where we obtained a total of 194 annotations.

**Fig. 10:**
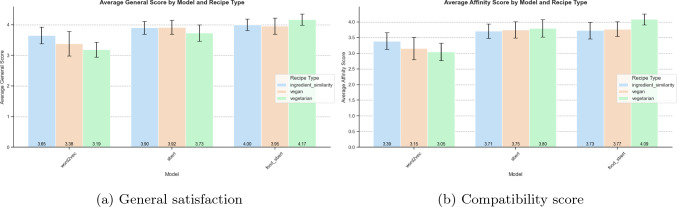
User satisfaction of the general recipe by model and adaptation type

**Fig. 11:**
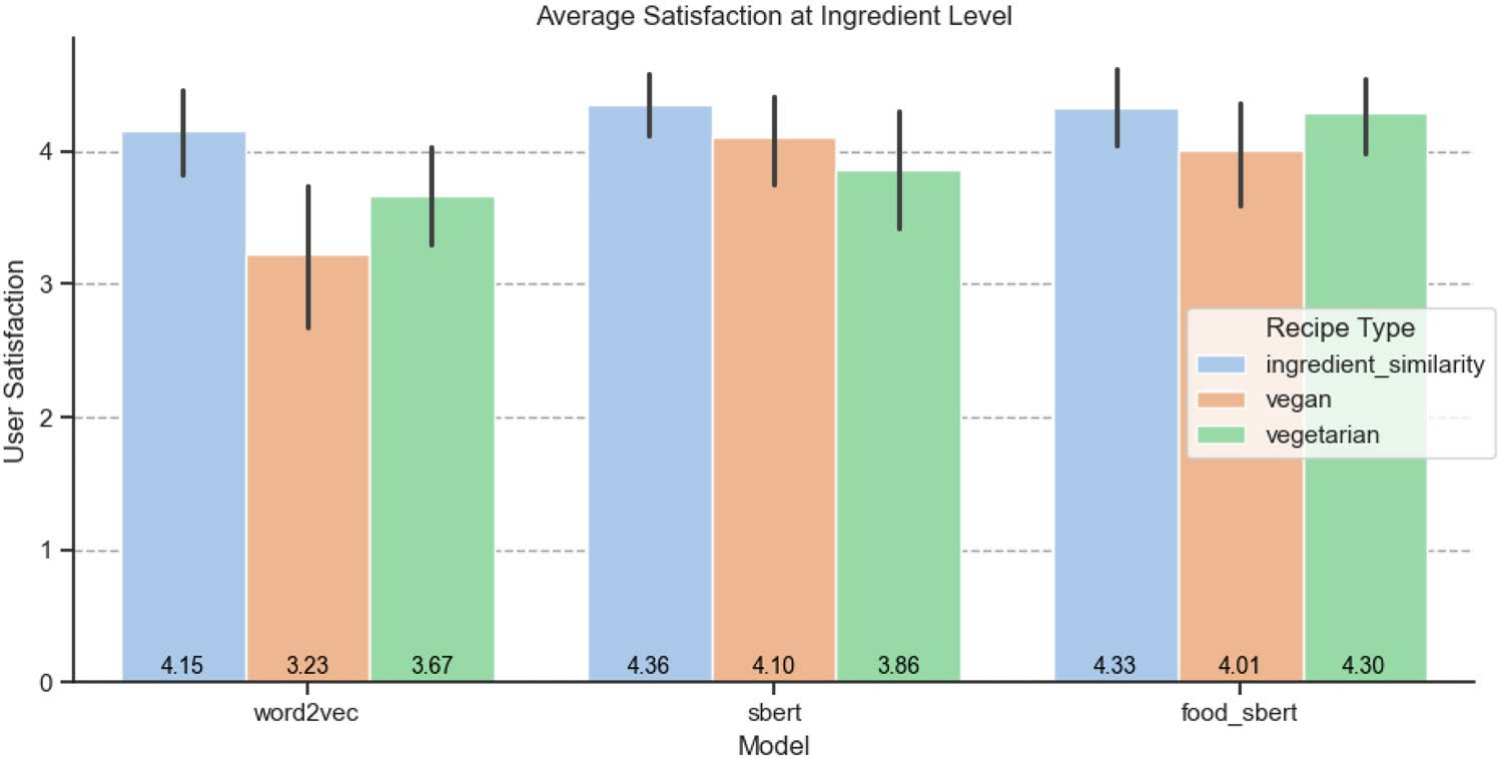
Average user satisfaction at ingredient level.

Regarding the user satisfaction at recipe level, Fig. 10a and b present the average scores across recipes for overall satisfaction and the compatibility of the adapted substitutions, respectively. At the ingredient level, we grouped annotations by the same ingredient, model, and restriction, calculating the average satisfaction value (see Fig. 11. Across both satisfaction levels, our results consistently exceeded a score of 3, highlighting the robustness and quality of our methodology regardless of the model used.

***Nutritional evaluation***

**Fig. 12:**
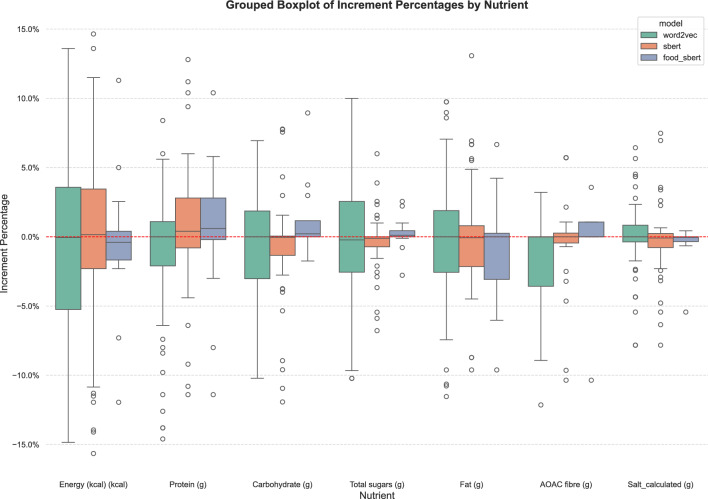
Nutritional variability of the models across nutrients

For the 100 most frequently used ingredients in the recipes from the human evaluation set, we analyzed the deviation in the nutritional profiles of their substitutions compared to the original ingredients. Figure 12 presents a visualization of the distribution of normalized increments for energy (kcal), protein, carbohydrates, sugar, fat, fibre, and salt. This information is shown for the general and re-trained SBERT models (referred to as *sbert* and *food_sbert* in the figure) as well as the Word2Vec implementation that preceded this study. The goal of this analysis is to identify which model minimizes overall percentage changes, aiming for increments close to zero.

**Fig. 13:**
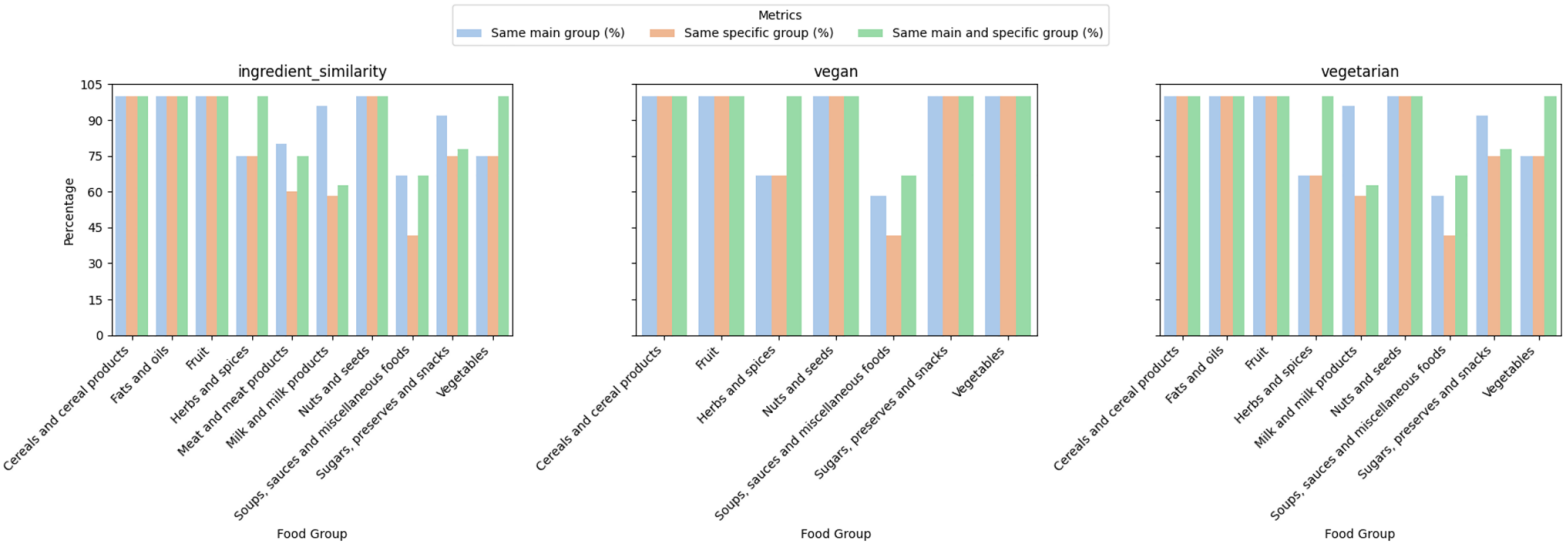
Study on the preservation of the original food main group and subgroups

Figure 13 illustrates the percentages of food group preservation in the recipes, categorized by the three types of adaptations considered. For each adaptation, we present three characteristics: the percentage of ingredients that preserve their main group among the top-5 best substitutions, the preservation of the same subgroup, and, among those that preserve the main group, the percentage that also shares the same subgroup.

***Ground-truth evaluation***

Table 10 shows the results obtained for the mention metrics for the pre-training and domain-specific SBERT modes, and the domain-specific word2vec.

**Table 10:** Evaluation of models based on precision, recall, F1-score at ingredient level, and regression metrics for analysing the embeddings

Model	Precision	Recall	F1-score	MSE	MAE
Domain-specific SBERT	**0**.**869**	**0**.**605**	**0**.**687**	**0**.**057**	**0**.**226**
Pre-training SBERT	0.360	0.163	0.217	0.205	0.421
Domain-specific word2vec	0.835	0.584	0.662	0.342	0.570

## Discussion

We have divided this discussion into the two main experimental parts of this work: behaviour and performance of (1) image captioning to extract the ingredients of the recipe and (2) recipe adaptation given the ingredient list.

### Image captioning

Detecting ingredients from images is challenging due to several reasons. We have listed below the more relevant and the analysis we have done consequently.

- Difficulty listing the ingredients from a recipe, where sometimes they are not explicitly distinguishable in the image. We have used BERTScore to study the similarity between the target and predicted captions.
- Difficulty determining, from an image, the number of ingredients used in a recipe. For this, we have studied the target and predicted the length of the generated captions.

Table 1 shows the similarity between the target and predicted captions. The results show that the three models achieve high accuracy, recall, and F1 when predicting the ingredient list regardless of the category. The performance achieved on the Food-101 dataset is both high and consistent across all metrics, which supports the robustness of the model in capturing ingredient information.

**Fig. 14:**
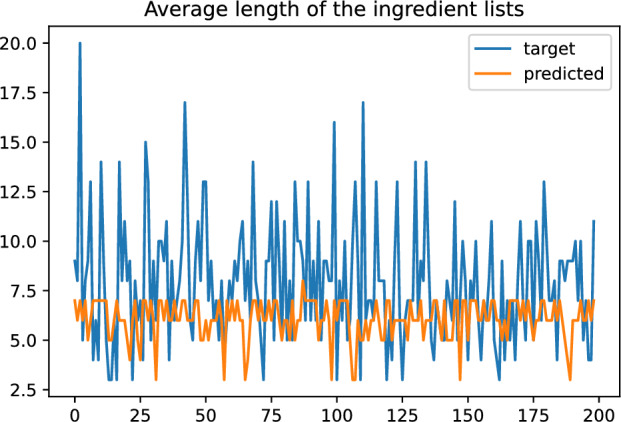
Length of target/predicted lists for the 199 recipes in validation

Figure 14 shows the difference between the length of target and predicted lists for the recipes of the validation set. It shows that the predicted ingredient lists tend to be short lists of ingredients (the average length of the predicted lists is 6.1 versus 7.9 of the target lists). Figure 7 is a representative example of this problem. It shows that the model struggles to detect fine-granulated and difficult to distinguish ingredients in the image, such as seasoning or spices. However, the model is able to detect whenever the recipe has more ingredients (it predicts more ingredients too) and vice-versa.

The findings underscore the distinct nature of dessert data and highlight the importance of category-specific training. As stated in Fig. 8a and 8a, the performance gap in desserts suggests that these recipes require unique modelling approaches to effectively capture their characteristics. The cross-category evaluations further demonstrate that models trained on appetisers or main dishes lack the adaptability needed for robust dessert performance.

The temperature analysis provided additional insights into model robustness. While variability introduced by higher temperatures enhanced performance for some categories, it negatively affected desserts, which may demand greater consistency (see Fig. 9). These results emphasize the need for tailored approaches in both model training and temperature tuning to optimize performance across diverse recipe categories.

### Recipe adaptation

In this experiment, we selected the 100 most common ingredients, from which we obtained the top-5 most similar foods in the external food dataset. It has been previously stated that one of our added difficulties is that we cannot ensure that the external food dataset will allow us to work with foods that can be easily found in food sets. For this reason, we refer to this metric as *lower bound*.

Regarding token-level representations obtained with BERT, Table 2 shows that the best results achieved for the lower bound are with the average of all the BERT hidden layers. Note that the results obtained with this strategy are quite different from those obtained from the other combinations, which are more alike. This means that the omission of the first layers leads to a loss of information in the encoding. It may be because our problem works with short descriptions. Therefore, it is not necessary to reach the level of the specification comprised of features of the last layers to be able to differentiate sentences. However, Table 2 also indicates that the outcomes obtained with BERT using all the hidden layers and SBERT (which only uses the last layer for token vectors) are similar. When studying sentence-level representations with SBERT (see Table 3), there are some relevant differences in the matching results obtained with the model, which uses max pooling strategy. This model gets the best results for this lower-bound. Mean-pooling and CLS representations generate embeddings of lower quality that lose preciseness when averaging token-level embeddings to build the sentence vector. We have concluded from our experiments that although using BERT with all the hidden layers may be the best option as a token-level strategy, the combination of SBERT using the last layer for token-level representation and the max pooling operation for sentence-level representation produces the best results. These results are consistent since the SBERT model is optimised for sentence representations.

Table 4 shows the results after re-training the SBERT model to the task domain. The values obtained for this lower bound are higher if we re-train the model to the food vocabulary. This is remarkable since we obtain greater results than the general model and our previous studies, despite achieving modest accuracy values. We attribute this to the difficulty of the fine-tuning task, which is hard to predict precisely due to many variations and styles in recipe texts. Since the food domain contains specific and closed vocabulary, re-training the model allows for obtaining more distinguishing and appropriate representations for food terms. Note that the chosen corpus also fits our final goal. We re-train the model with texts from the cooking instructions. These texts contain information regarding which ingredients usually appear in the same recipes, which are mixed and cooked together in a specific step, and also information regarding cooking procedures or dressings. It is the kind of data we pursue to encode in a food vector representation when the final purpose is to detect food replacements and semantic substitutions and relationships.

From the human evaluation study, the results illustrate that among the models, the one retrained for the food domain (food_sbert, as shown in the figure) achieved the best overall results, as shown in Fig. 10a. Its average scores for each adaptation type exceeded 4, with less variation across adaptation types, demonstrating its reliability and effectiveness. The user satisfaction at the ingredient level is consistent (see Fig. 11), showing overall good performance and overperforming the model retrained to the food domain, as it achieves a high while stable user satisfaction scores.

The human evaluation of the compatibility score in Fig. 10b, illustrates the behaviour is very similar to that observed for general satisfaction, achieving average scores above 3. This reflects the strong performance of our approach in terms of compatibility. Notably, the word2vec model demonstrates lower consensus among annotators for the vegan restriction.

Regarding the nutritional variations of the substitutions, as illustrated in the figure, word2vec appears less suitable due to higher variability in key nutrients such as carbohydrates and sugar. In contrast, the SBERT models demonstrate distributions more centred around 0% for most nutrients. The re-trained SBERT model offers a balance between minimizing variability and maintaining consistency across nutrients. Overall, Fig. 12 highlights the re-trained SBERT model as the most balanced in terms of nutritional consistency relative to the original ingredients. Its tighter distributions across most nutrients indicate a high level of consistency and minimal extreme variations, which are crucial when considering nutrition-related modifications. With respect to the differences between original and substitute food groups, the results reveal that the level of preservation across all groups is generally high. However, preservation tends to be lower in groups with greater ingredient variability, such as *Soups, Sauces, and Miscellaneous foods* (see Fig. 13). This finding is expected due to the broad scope of dishes included in that category compared to more narrowly defined groups, such as meat products or vegetables.

We have achieved high-quality results in the experiments with the ground-truth dataset (see Table 10), particularly with the retrained embeddings tailored to food data, demonstrating their ability to encode nuanced food knowledge. This is especially evident when analysing massive data resources that extend beyond commonly used recipe datasets with frequent ingredients, as in the case of the ground truth that we have used. These results emphasize the embeddings’ capacity to generalize and capture the complexity of food substitution, even in less conventional contexts. However, while this approach is practical for evaluating adaptation quality, it should not be solely relied upon for two reasons. First, food substitution is inherently subjective, as what constitutes a suitable substitute can vary significantly depending on individual preferences and cultural backgrounds. Second, the task is highly recipe-dependent; an ingredient that works as a substitute in one recipe might not be appropriate in another due to the intricate interplay of ingredient combinations and cooking procedures. This underscores the importance of considering user satisfaction for evaluation and studying nutrition variation to ensure that substitutions maintain nutritional balance regarding the originals.

**Fig. 15:**
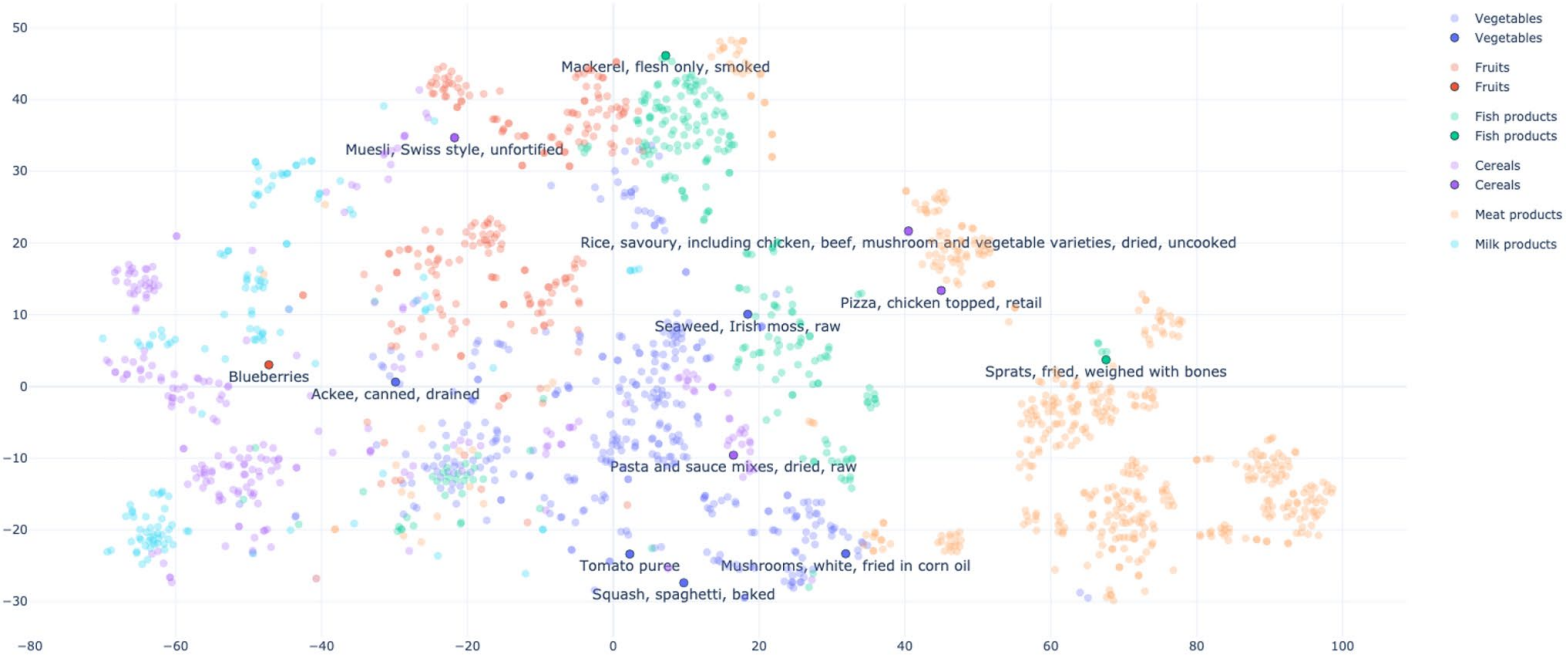
A visualisation of the embeddings from the CoFID foods

Figure 15 illustrates the influence of these relationships between food items when building the embeddings. It shows the latent space with the food item embeddings from the CoFID table. Each dot corresponds to a food item from the table and its colour to the food category they belong to. Specifically, it shows food items from the categories with more than 100 items (i.e., vegetables, fruits, cereals, fish, milk, and meat products.). Some appear highlighted to show relevant features captured by the embeddings obtained with the domain-specific model. The “blueberries” embedding (left side of the figure) appears near the milk and cereal products and not on other fruits since it stands out for being used together with these kinds of products. It is a similar case for “tomato purée” and “mushrooms, white, fried in corn oil”, which usually appear combined with pasta, and “Squash, spaghetti, baked” a vegan substitute for pasta (bottom-side of the figure). Similarly, items such as “pizza, chicken topped, retail” and “rice, savoury, including chicken, beef, mushroom...” appear far from the rest of the elements of their category (i.e., cereals) but near to meat products, since they are usually cooked with them. These encoded features are helpful for recipe adaptation since they help detect food item combination/modification relations.

**Fig. 16:**
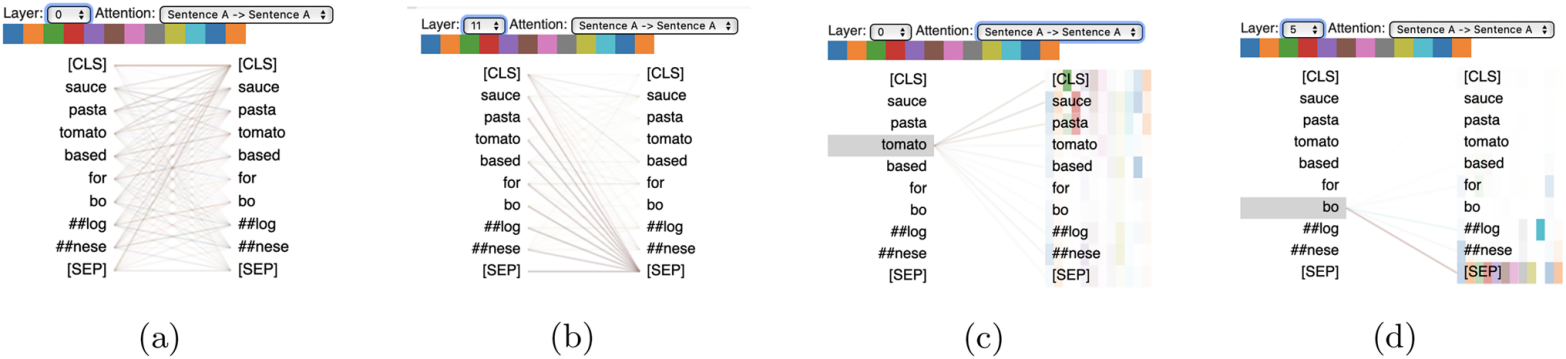
Bertviz [54] attention visualisations with using “sauce pasta tomato-based for bolognese” as input. It shows attention-head information of the input sequence (note that the tokens represent minimum-meaning words, and some can be split into more than one token, such as “bolognese”). It illustrates: (a) attention-head view for layer 0; (b) attention-head view for layer 11; (c) attention-head view with the token “tomato” selected (layer 0); (d) attention-head view with the token “bo” selected (layer 5)

Regarding the behaviour of the domain-specific model for the recipe adaptation task, Tables 5 and 6 show the results obtained with general-purpose and domain-specific SBERT models adapting the same recipe, respectively. They illustrate that the results obtained with the re-trained model in the food domain are more accurate. Table 6 shows that the model allows for reaching options maintaining the main food ingredient in the description, e.g., see results for ingredients “Sour cream”.

Table 7 shows the suggested food replacements obtained for adapting the same recipe using the recipe adaptation method proposed on [4] (no restrictions considered). The results illustrate that the performance of AdaptaFood is better since the model can prioritise the main ingredient in the suggestions. We also observed that contrary to AdaptaFood, the method from [4] obtains some inconsistencies, e.g., for sour cream, it suggests “piccalilli” (a relish) and an alcoholic drink. Here we highlight the role of attention mechanisms in measuring the token relevance for item-matching purposes. Figure 16 shows the attention values for the food description “sauce pasta tomato-based for bolognese” obtained with Bertviz [54]. This tool visualises self-attention as lines linking the tokens that are attending (left) with those being attended to (right). The line weight represents the attention score/relevance between these tokens. It illustrates, for example, the tokens influenced by “tomato” and its degree of relevance, thus giving a measure of what is relevant in the sequence. Considering this additional information allows for better-quality vectors for short-document semantic tasks. This fact is engaging in our problem since one issue when working with datasets from heterogeneous sources is the need to address the difference in the detail level in the text descriptions. This difference appears in many ways: very detailed but irrelevant for the same food item, information regarding peeling for vegetables or fruits, and information regarding the style of cutting for meat, amongst others. This issue does not affect the recipe adaptation task output but should be considered in the model encoding to work jointly with the datasets.

With AdaptaFood, we can add as many dietary requirements as required. Apart from food similarity for adapting recipes, we show an example of vegetarian restrictions (see Table 8). From these results, we can observe that the quality of the food replacements depends on the variety and diversity of foods from external resources. For example, having a vegan-related food dataset with various vegan-based foods, such as meat substitutes, would provide more accurate vegan food replacements. Note the food replacements also obtained in Table 8. The vegetarian option for “bacon” suggests some reflections. AdaptaFood proposes to use cheese instead of bacon for obtaining a vegetarian recipe, which seems a suitable replacement since bacon and cheese appear together in many dishes. Table 9 shows vegan adaptations for the same ingredients as Table 8. In this case, AdaptaFood suggests changing “bacon” for bread-related ingredients, which are also ingredients that can appear together with bacon. General food datasets (as is the case of CoFID) do not usually contain vegan-specific products from the market, which prevents the model from suggesting more sophisticated vegan substitutes.

One of the main difficulties is the lack of labelled datasets for recipe adaptation. Many studies suggest ingredient alternatives by creating a customised ground truth of possible suggestions for the ingredients [12]. This is complex since it is difficult to find a representative subset of foods able to extrapolate and represent food recipes in general. We plan to build an extensive food suggestion dataset to properly develop this kind of evaluation, focusing on dietary requirements and healthy diets. Adapting a recipe is one of many tasks we can perform with AdaptaFood, e.g., we could use it to extrapolate nutrition data from the ingredients to obtain an estimation of the nutritional information of the recipe. However, it may be used for other domain-specific tasks, e.g., for adapting expert recommendations in healthcare, recipes and preparation steps from materials or commercial products. By this, we mean that any recipe/structured object can be a candidate to be modified to satisfy specific characteristics with our adaptation approach by changing the datasets to the matter domain.

## Conclusion

AdaptaFood permits the automatic and massive adaptation of recipes, thus taking advantage of existing resources without creating whole new recipes from scratch. The results show that using attention vectors when building sentence representations generates quality representation embeddings for food descriptions, especially when using the max pooling strategy. This is due to the fact that the vectors ponder the relevance of each token in the sentence, thus tackling the difference in the detail in food item descriptions from different resources. Re-training the model to the food domain improves these representations and better matches food items amongst datasets. This has been demonstrated through the human, nutrition, and ground-truth experiments, which highlight the remarkable performance of AdaptaFood across dietary restrictions, particularly when utilising the domain-specific language model. We plan to incorporate user profiles (e.g., preferences and/or medical records) and design a workflow to adjust ingredient quantities. We also aim to consider regional facts to fulfil user requirements better and incorporate biochemical information regarding flavours and food pairings to enrich the adaptation.

AdaptaFood also enables the connection of several datasets from different sources. In our case, we have related one source with food names (i.e., the ingredient list from the recipe) to a knowledge-specific nutrition database. This task is of prime relevance since it allows us to extend the recipe information. The multi-modality of AdaptaFood allows us to adapt recipes from different formats. We plan to add expert knowledge and implement a specific metric to enhance the performance of the trained image-captioning module. Handling heterogeneous and incomplete data is an ongoing challenge, specially when AdaptaFood expands to new external resources. Future iterations of the system will incorporate machine learning models to predict missing values based on ingredient similarities and expand the nutritional database to enhance substitution reliability.

## Data Availability

The data used for this study is openly available and we provide the corresponding links to data repositories thorough the paper.

## Footnotes

1 Diabetes statistics provided by WHO: https://www.who.int/news-room/fact-sheets/detail/diabetes.

2 Link: https://www.oxfordmartin.ox.ac.uk/news/201603-plant-based-diets/.

3 Supported by the European Union under grant agreement No. 816303.

4 The code, trained models, and data (or links to the sources instead) will be publicly available on GitHub upon acceptance of the paper.

5 EuroFIR: https://www.eurofir.org.

6 https://www.bbc.co.uk/food.

7 https://www.kaggle.com/kaggle/recipe-ingredients-dataset.

8 https://www.kaggle.com/nehaprabhavalkar/indian-food-101/version/2.

9 https://www.kaggle.com/sarthak71/food-recipes.

10 https://www.gov.uk/government/publications/composition-of-foods-integrated-dataset-cofid.

11 https://www.kaggle.com/datasets/shuyangli94/food-com-recipes-and-user-interactions.

12 Pre-trained BERT model: https://huggingface.co/bert-base-uncased.
